# Fitness response variation within and among consumer species can be co-mediated by food quantity and biochemical quality

**DOI:** 10.1038/s41598-019-52538-2

**Published:** 2019-11-06

**Authors:** Svenja Schälicke, Johannes Teubner, Dominik Martin-Creuzburg, Alexander Wacker

**Affiliations:** 1 Institute of Biochemistry and Biology, University of Potsdam, Am Neuen Palais 10, D-14469 Potsdam, Germany; 2 Limnological Institute, University of Konstanz, Mainaustr. 252, D-78464 Konstanz, Germany; 3 Animal Ecology group, Zoological Institute and Museum, University of Greifswald, Loitzer Str. 26, D-17489 Greifswald, Germany

**Keywords:** Freshwater ecology, Limnology, Ecophysiology, Biodiversity

## Abstract

In natural heterogeneous environments, the fitness of animals is strongly influenced by the availability and composition of food. Food quantity and biochemical quality constraints may affect individual traits of consumers differently, mediating fitness response variation within and among species. Using a multifactorial experimental approach, we assessed population growth rate, fecundity, and survival of six strains of the two closely related freshwater rotifer species *Brachionus calyciflorus* sensu stricto and *Brachionus fernandoi*. Therefore, rotifers fed low and high concentrations of three algal species differing in their biochemical food quality. Additionally, we explored the potential of a single limiting biochemical nutrient to mediate variations in population growth response. Therefore, rotifers fed a sterol-free alga, which we supplemented with cholesterol-containing liposomes. Co-limitation by food quantity and biochemical food quality resulted in differences in population growth rates among strains, but not between species, although effects on fecundity and survival differed between species. The effect of cholesterol supplementation on population growth was strain-specific but not species-specific. We show that fitness response variations within and among species can be mediated by biochemical food quality. Dietary constraints thus may act as evolutionary drivers on physiological traits of consumers, which may have strong implications for various ecological interactions.

## Introduction

Functional traits shape the fitness response of an organism to environmental changes by directly affecting components of the individual performance, such as growth, reproduction, and survival^1^. Thus, traits are also supposed to crucially affect interactions among species, such as competition or trophic relationships^2–4^. Individuals of one species have long been treated as ecologically equivalent. However, variations in individual traits and in corresponding fitness responses to environmental changes exist not only between, but also within species^5^. This emphasizes the need of functional trait-based approaches in ecology rather than focussing on taxonomic identities^1,6,7^.

The population growth rate is a key fitness measure comprising all components of individual performance. Thus, it links functional traits to responses on the community level^3^ and is therefore frequently used to evaluate interactions within and across trophic levels^4,8^. The population growth response of a consumer is crucially influenced by the quantity and the quality of the available prey. Food quantity is defined as the amount of carbon providing energy for the consumer. Food quality is more complex, as it comprises morphological prey traits influencing the processing of food items as well as nutritional traits acting on the consumer’s physiology. In natural systems, food quantity and biochemical quality is highly variable, potentially resulting in dietary constraints consumers have to cope with^9,10^. This is especially relevant at the zooplankton-phytoplankton interface in aquatic systems, where many unselective filter feeders, primary consumers such as cladocerans or rotifers, strongly depend on the dietary nutrient supply.

Intraspecific trait variation in response to food quantity^11,12^, elemental food quality^13^, or a combination of both^14^ has been studied already, mainly in aquatic consumers. However, besides elemental nutrients, the performance of a consumer can be limited also by essential biochemical nutrients, such as polyunsaturated fatty acids (PUFA)^9,15^, sterols^16,17^ or certain amino acids^17,18^. Evidence for lipid-mediated fitness response variation within consumer species is scarce (but see^19–21^). Intraspecific response variation represents a major target for processes of natural selection. Linking resources to variations in performance components could thus help to identify drivers of niche differentiation and could improve predictions of ecological interactions and community dynamics.

Rotifers of the genus *Brachionus* are frequently used in trait-based ecological studies^4,8,22–24^. As unselective filter feeders^25^, they are highly susceptible to food quantity and quality limitations. The dietary availability of sterols, PUFA, and certain amino acids has been shown to strongly affect different performance components of rotifers, such as population growth rate, egg production, fecundity and survival^17,18,26^. Recently, four species of *Brachionus calyciflorus* have been (re)described based on phylogenetic and morphometric differences among strains within this species complex^27^. These species have been shown already to differ in their life history^28^ and in their heat stress tolerance^29^, potentially driving natural selection and thus the evolution of new genotypes^30^.

Here, we experimentally studied how food quantity and biochemical food quality limitation affect fitness response variation among *Brachionus* strains and species. We used six strains of two closely related and recently (re)described *Brachionus* species (*B*. *calyciflorus* sensu stricto (s.s.) and *Brachionus fernandoi*, three strains each) and assessed rotifer’s susceptibility, i.e. the change in population growth rate, fecundity and survival under different dietary nutrient supplies. We let them feed on high and low concentrations of three algal species (*Nannochloropsis limnetica*, *Monoraphidium minutum* and *Synechococcus elongatus*) differing mainly in their biochemical food quality. We furthermore explored the potential of a single limiting biochemical nutrient, i.e. cholesterol, to mediate variations in population growth response. Therefore, the rotifer strains fed *S*. *elongatus*, a sterol-free alga, which was supplemented with cholesterol-containing liposomes. We hypothesized that response variations among *Brachionus* strains and species are mediated by food quantity and biochemical food quality.

## Results

**Figure 1:**
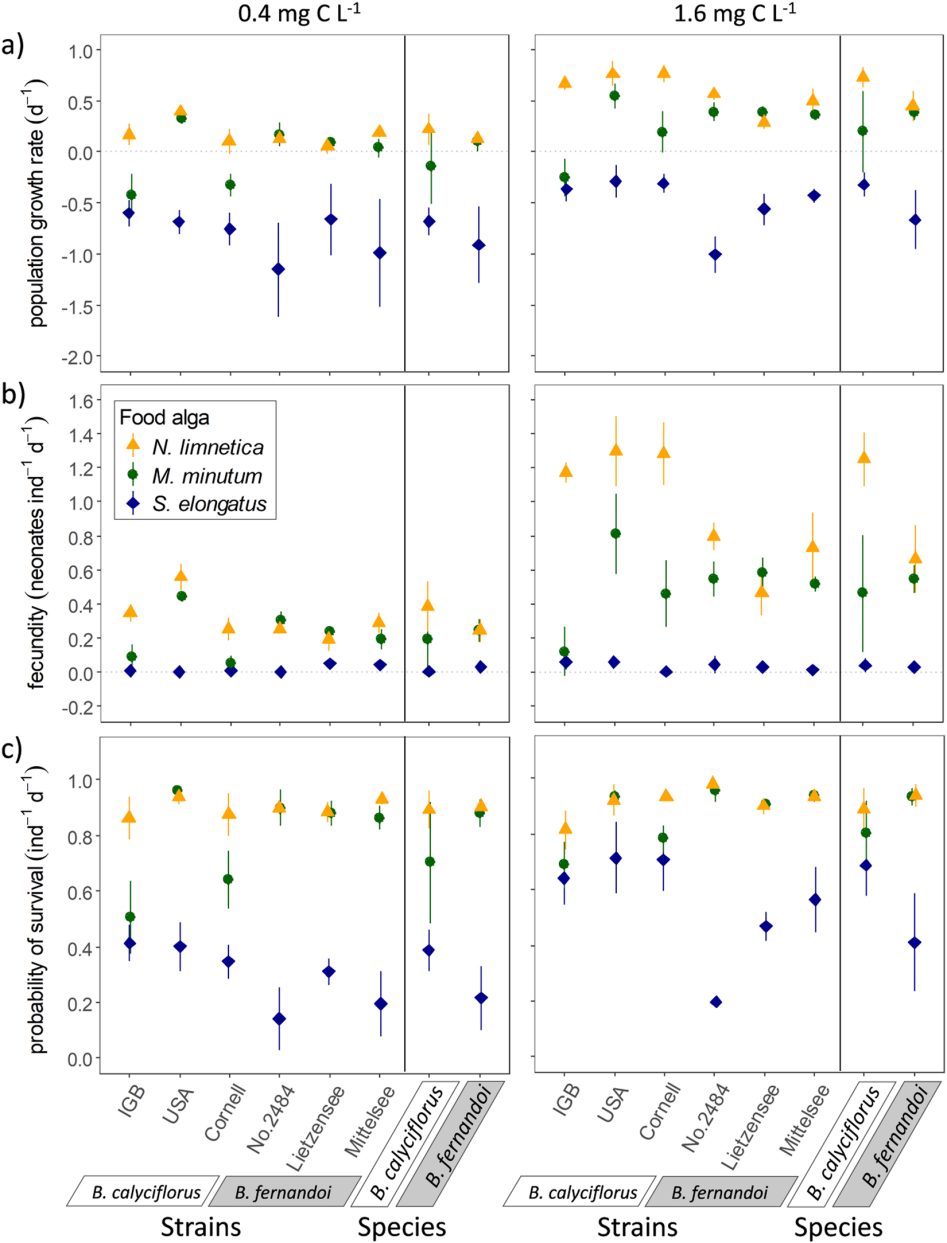
Population growth rates, fecundity and probability of survival of six strains of the two rotifer species *Brachionus calyciflorus* s.s. (‘IGB, ‘USA’, ‘Cornell’) and *Brachionus fernandoi* (‘No. 2484’, ‘Lietzensee’, ‘Mittelsee’) feeding on one of the three unicellular algae species *Nannochloropsis limnetica*, *Monoraphidium minutum* and *Synechococcus elongatus*. Each food alga was supplied in two quantities (0.4 and 1.6 mg C L^−1^). Values represent means ± standard deviation. For strains, the number of replicates was usually N = 4 with five exceptions in the population growth rates with N = 3 (IGB and Cornell fed 1.6 mg C L^−1^ *M*. *minutum*; No. 2484, Lietzensee, and Mittelsee fed 0.4 mg C L^−1^ *S*. *elongatus*). For species, all replicates of the respective strains were included for the calculation, resulting in N = 72 for fecundity and survival and N = 70 and N = 69 for population growth rates of *B*. *calyciflorus s*.*s*. and *B*. *fernandoi*, respectively.

In the first experiment, population growth rates of rotifers were affected by the type of food alga provided, characterized mainly by differences in their lipid composition, as well as by the food quantity, i.e. the carbon concentration (Table 1, Fig. 1a). The magnitude of these effects differed between the two rotifer species, indicated by significant interactions in the ANOVA (interaction, food alga × species, F_2,103_ = 47.2, *P* < 0.001, interaction, food quantity × species, F_1,103_ = 13.7, *P* < 0.001, Table 1). The missing three-way interaction among food quantity, food quality and species (F_2,103_ = 1.6, *P* = 0.20; Table 1) indicates that the species-specific pattern in the population growth response to the food algae did not change from low to high food quantity. Within each rotifer species, a high response variation was recognized, i.e. population growth rates in response to different food quantities and qualities were highly strain-specific (nested three-way interaction, F_24,103_ = 11.6, *P* < 0.001; Table 1).

**Table 1:** Results of nested three-way ANOVAs using Box-Cox-transformed data on population growth rates and fecundity and arcsine-transformed data on survival of strains nested within the two rotifer species *Brachionus calyciflorus* s.s. and *Brachionus fernandoi*. Six strains, three of each species, were provided with two quantities (0.4 and 1.6 mg C L^−1^) of three unicellular algae (*Synechococcus elongatus*, *Monoraphidium minutum*, *Nannochloropsis limnetica*) of different biochemical quality.

Independent variable(s)	Population growth rate (d^−1^)			Fecundity (neonates ind^−1^ d^−1^)			Probability of survival (ind^−1^ d^−1^)		
	*df*	*F*-value	*P*-value	*df*	*F*-value	*P*-value	*df*	*F*-value	*P*-value
Food quantity (FQ)	1, 103	245.3	<**0**.**001**	1, 108	340.0	<**0**.**001**	1, 108	73.8	<**0**.**001**
Food alga (FA)	2, 103	851.4	<**0**.**001**	2, 108	642.4	<**0**.**001**	2, 108	544.3	<**0**.**001**
Species (Sp)	1, 103	17.1	<**0**.**001**	1, 108	10.1	<**0**.**01**	1, 108	1.6	0.20
FQ × FA	2, 103	10.1	<**0**.**001**	2, 108	80.3	<**0**.**001**	2, 108	13.6	<**0**.**001**
FQ × Sp	1, 103	13.7	<**0**.**001**	1, 108	14.7	<**0**.**001**	1, 108	0.03	0.86
FA × Sp	2, 103	47.2	<**0**.**001**	2, 108	59.3	<**0**.**001**	2, 108	65.9	<**0**.**001**
FQ × FA × Sp	2, 103	1.6	0.20	2, 108	8.2	<**0**.**001**	2, 108	4.8	<**0**.**01**
FQ × FA × Sp × Strain	24, 103	11.6	<**0**.**001**	24, 108	9.9	<**0**.**001**	24, 108	8.9	<**0**.**001**

Rotifer strains responded differently to food quantity and quality also regarding fecundity (nested ANOVA, three-way interaction, F_2,108_ = 9.9, *P* < 0.001; Table 1) and survival (F_2,108_ = 8.9, *P* < 0.001). In contrast to population growth rates, however, we additionally found species-specific responses to food quantity and quality regarding fecundity (ANOVA, three-way interaction, F_2,108_ = 8.2, *P* < 0.001, Table 1) and survival (F_2,108_ = 4.8, *P* < 0.01).

Especially apparent at high food quantity, the fecundity of *B*. *calyciflorus* s.s. was more susceptible to food quality (increase of 1.2 neonates ind^−1^ d^−1^, Fig. 1b) than that of *B*. *fernandoi* (increase of 0.6 neonates ind^−1^ d^−1^), as indicated by a broader range in the response from low-quality (*S*. *elongatus*) to high-quality (*N*. *limnetica*) food. In contrast, considering the probability of survival, *B*. *fernandoi* was more susceptible to food quality (increase of 0.5 d^−1^, Fig. 1c) than *B*. *calyciflorus* s.s. (increase of 0.2 d^−1^). All species-specific as well as strain-specific susceptibilities to food quality are provided in the Supplementary Information 2.

The magnitude of response variation among rotifer strains and species was strongly dependent on the type of food. For example, at high food quantity, fecundity response variation was stronger on high-quality *N*. *limnetica* (variation range of 0.58 neonates ind^−1^ d^−1^, Fig. 1b) than on low-quality *S*. *elongatus* (range of 0.01 neonates ind^−1^ d^−1^). In contrast, regarding the probability of survival, the response variation was stronger on *S*. *elongatus* (variation range of 0.27 d^−1^, Fig. 1c) than on *N*. *limnetica* (range of 0.04 d^−1^).

**Figure 2:**
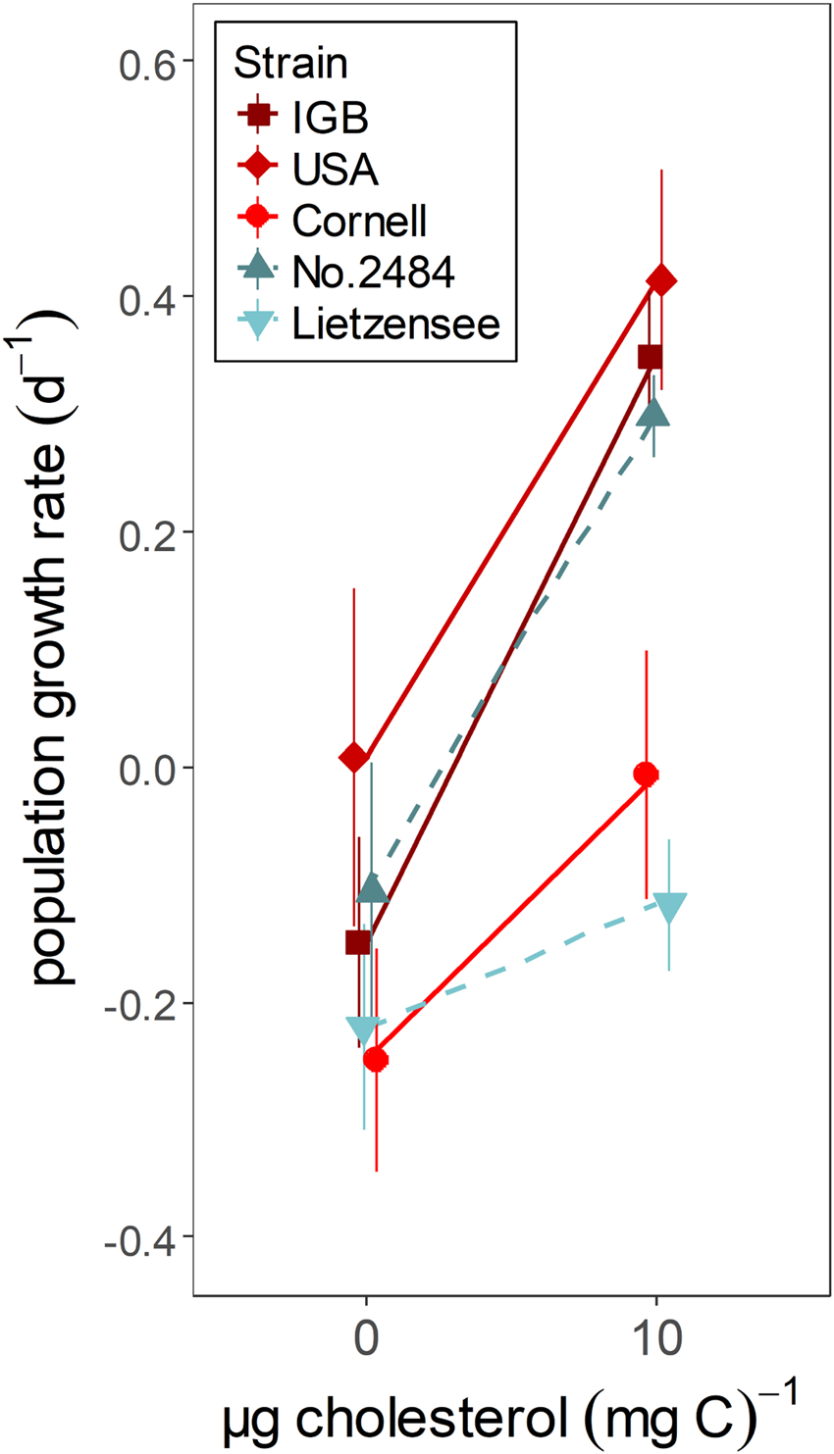
Population growth rates of five strains of the two rotifer species *Brachionus calyciflorus* s.s. (‘IGB, ‘USA’, ‘Cornell’; red symbols and solid lines) and *Brachionus fernandoi* (‘No. 2484’, ‘Lietzensee’; cyan symbols and dashed lines) feeding on sterol-free *Synechococcus elongatus* (3.2 mg C L^−1^) supplemented with liposomes with cholesterol content (10 µg cholesterol (mg C)^−1^) or without. Values represent means ± standard deviation, number of replicates N = 5.

In the second experiment, population growth rates of rotifers feeding on sterol-free *S*. *elongatus* increased significantly upon cholesterol supplementation (two-way ANOVA, factor cholesterol, F_1,40_ = 68.0, *P* < 0.001; Fig. 2). This cholesterol effect, and thus rotifers’ susceptibility to dietary cholesterol, did not differ between species (interaction, F_1,40_ = 0.8, *P* = 0.36), but differed significantly among strains (nested two-way ANOVA, interaction, F_6,40_ = 29.1, *P* < 0.001).

## Discussion

Here, we highlight the potential of biochemical food quality constraints to mediate fitness differences among rotifer strains and species. We show experimentally that food quantity and quality interactively affect population growth rates of *Brachionus*. This susceptibility, however, differed among strains but not between the two species *B*. *calyciflorus* s.s. and *B*. *fernandoi*. The ingestion rates with which the different food algae were consumed did not differ among rotifer strains (Supplementary Information 3), suggesting that the observed response variation was not mediated by morphological traits of the food algae. The three microalgae species we used as food slightly differed in their phosphorus content (Table 2), but all revealed non-limiting P supply for rotifers^31,32^. However, the algae differed substantially in their biochemical nutrient composition (Table 2). Therefore, we assume that the response variation among rotifer strains and species was mediated by the availability of essential biochemicals, such as sterols and PUFA. This assumption could be confirmed in a second experiment, in which we manipulated the biochemical food quality of an algal prey by supplementing cholesterol, a sterol that has been shown previously to improve population growth rates of *Brachionus* at low dietary sterol supply^17,26^. Supplementation of *S*. *elongatus* with cholesterol significantly increased population growth rates. Comparable to our first experiment, this response to food quality was strain- but not species-specific. Considering the population growth response of *B*. *calyciflorus* s.s. and *B*. *fernandoi* strains to biochemical food quality, our results suggest a higher fitness response variation within than between species.

**Table 2:** Food quality characteristics of *Synechococcus elongatus*, *Monoraphidium minutum* and *Nannochloropsis limnetica* used as food algae for *Brachionus*.

	*S. elongatus*	*M. minutum*	*N. limnetica*
Size (equivalent spherical diameter)	1 µm	3.5 µm	<3 µm
Shape	oblong	arcuate	spherical
C:N:P [molar]	113.9:14.9:1	21.8:2.6:1	87.7:9.4:1
**Sterols [µg (mg C)** ^**−1**^ **]**			
total	nd	14.0	16.9
Cholesterol	nd	nd	11.1
25-Dehydrochondrillasterol	nd	5.3	nd
5α-Ergosta-7,25-dien-3β-ol	nd	3.9	nd
Isofucosterol	nd	nd	3.1
24-Methylenecholesterol	nd	nd	1.5
5α-Poriferasta-7,25-dien-3β-ol	nd	1.4	nd
24-Methylenelathosterol	nd	1.1	nd
**Fatty acids [µg (mg C)** ^**−1**^ **]**			
total	73.1	258.0	189.1
SFA	40.3	64.1	60.7
MUFA	32.9	105.2	88.8
PUFA	nd	88.7	39.6
C18:2n-6	nd	10.7	2.4
C18:3n-6	nd	nd	1.3
C20:4n-6	nd	nd	5.6
C18:3n-3	nd	51.4	0.7
C18:4n-3	nd	9.4	nd
C20:5n-3	nd	nd	25.3

*Note:* Sizes were taken from Rothhaupt^25^ (*S*. *elongatus* and *M*. *minutum*) and from Krienitz and Wirth^49^ (*N*. *limnetica*). Sterols and PUFA with a concentration higher than 1 µg (mg C)^−1^ are listed. SFA represent all saturated fatty acids with ≥14 carbon atoms; MUFA represent all fatty acids with a single double bond ≥16 carbon atoms; PUFA contain all fatty acids with at least two double bonds and ≥18 carbon atoms. C:N:P, sterol and fatty acid data of *S*. *elongatus* and *N*. *limnetica* were taken from Schälicke *et al*.^26^, because the same algal chemostat cultures were used in this study. Chemical analysis was conducted as described in Schälicke *et al*.^26^. Data represent means of duplicates, nd = not detected.

Differences in population growth rates among rotifer strains feeding on different food algae have been reported previously^33–35^. In none of these studies, however, the algal trait mediating the variation in population growth responses among strains has been investigated. Brzeziński and von Elert^20^ provided evidence for differences in somatic and population growth responses to dietary PUFA supplementation among *Daphnia* clones, emphasizing the significance of essential biochemicals in mediating intraspecific fitness variation.

Most animals, including rotifers, have no or only restricted abilities to synthesize essential lipids, such as sterols and PUFA, *de novo* and therefore rely on an adequate dietary supply to cover their physiological demands^36–38^. However, experiments with rotifers provided evidence that they are able to actively elongate and desaturate long-chain PUFA from dietary precursors^17,39,40^. As a supplementation of a single PUFA to a lipid-poor food algae did not have a significant effect on population growth of rotifers, a simultaneous limitation (co-limitation) of their growth by different biochemicals is highly likely^41^. This is supported by a recent study, which demonstrated that exchanging small proportions of a PUFA- and sterol-free alga by a lipid-rich alga increased the population growth rate of *B*. *calyciflorus* much stronger than a supplementation of cholesterol to the lipid-poor algal food^26^.

Biochemicals take up crucial tasks in different biological processes of an individual. Sterols, for instance, are important structural components of cell membranes and thus may be important primarily for somatic growth^41^. Fatty acids are energy-rich storage compounds that are eventually catabolized to fuel metabolic processes, which is critical for maintenance and survival. Furthermore, fatty acids are required as structural components in cell membranes and for reproduction-related processes^18,42^. Hence, a limitation by essential lipids could exert distinct selection pressure on various physiological traits affecting distinct components of individual performance. In our experiments, fecundity and survival were differently affected by food quantity and quality co-limitation and both responses revealed strong intra- and interspecific variation. Variation in fecundity was strongest when rotifers fed on the lipid-rich food *N*. *limnetica*. This suggests that lipids required for reproduction-related processes, such as PUFA provided by *N*. *limnetica*, were utilized differently by the rotifer strains and species. However, when they fed on *S*. *elongatus*, which lacks essential lipids required for reproduction, the response variation in their fecundity was low. Contrasting food quality effects on response variation were found for survival. *Brachionus* showed a high response variation in survival when feeding on *S*. *elongatus*, which is deficient in essential lipids but nonetheless provides carbon for maintenance. The rotifer strains and species we used here may differ in physiological traits affecting the storage and allocation of resources required for survival. On high-quality *N*. *limnetica*, survival of rotifers was generally high with low response variation. A strong response variation may indicate that biochemical food quality, in interaction with food quantity, facilitates niche separation of consumers. At low food quality, selection may act primarily on survival-related traits, while at high food quality, the availability of various resources might open up niches for adaptations in fecundity-related traits to maintain high fitness.

Trade-offs are among the most plausible mechanisms for trait variations, as they fundamentally reduce the individual niche widths to minimize competition^5^. Under resource limitation, rotifers use different strategies to cope with the trade-off of allocating resources either into reproduction-related or survival-related processes to maintain population growth^26,43,44^. Evolution may thus act on this trade-off when rotifers are co-limited by food quantity and biochemical food quality. The strain- and species-specific differences in response variations we observed in *Brachionus* might relate to variations in physiological traits, such as resource storage or allocation, resulting in strain- and species-specific strategies to maintain high population growth rates. Comparably, Ricci^34^ found strain-specific effects on reproduction and survival within a rotifer species feeding on different algae, which the author related to strain-specific life history strategies.

Biochemical nutrients can act as drivers of fitness response variation among strains and species, which is suggested by the results presented here for *Brachionus* and by those of Brzeziński and von Elert^20^ for *Daphnia*. Brzeziński and von Elert^20^ analysed the susceptibility of growth rates of different *Daphnia* clones to the limitation by a single PUFA. We show here that, besides PUFA, also sterols, e.g. cholesterol, can drive performance differences among strains and species of a freshwater herbivore.

Inter- and intraspecific variations in fitness responses, as we observed in our study, can be attributed to individual traits of organisms. How these functional traits are affected by environmental changes is determined genetically. The rotifer strains we used were isolated from different lakes and variations in performances might be the consequence of specific adaptations to available resources in their respective habitats or adaptations to avoid local resource competition. Comparable to stoichiometric food quality^13,14^, biochemical food quality limitations may exert selection pressure on natural species assemblages. This might involve adaptations in physiological traits (e.g. nutrient absorption and bioconversion capacities) and requirements, which eventually manifest genetically^30^.

We show here experimentally that food quantity and biochemical food quality co-mediate fitness response variation within two herbivorous zooplankton species. This suggests that lipid-mediated food quality constraints exert selection pressure on *Brachionus* populations. Potential physiological trait adaptations to food quality may increase the functional diversity within species and may open possibilities for niche separation, coexistence and the evolution of new genotypes. We found a higher variation in population growth responses within than between species. Thus, species-specific fitness responses should only cautiously be used as tool to assess community dynamics, as they strongly depend on the functional trait diversity that the considered species comprises. Further studies are required, using different strains of a species, to identify trade-offs, traits and the underlying mechanisms involved in food-related response variations. Analysing genes that are responsible for physiological adaptations and trait variations may help to elucidate the genetic background of response variation within species. Studying intra- and interspecific response variations of consumers on the individual and population level may help specifying the functional niche of consumers, which in turn would improve trait-based approaches assessing community dynamics and predator-prey interaction in both terrestrial and aquatic systems.

## Methods

### Cultivation of organisms

In the experiments, we used six asexually reproducing strains of the genus *Brachionus*; three strains of *B*. *calyciflorus* s.s. (‘IGB’; ‘Cornell’; ‘USA’) and three strains of *B*. *fernandoi* (‘No. 2484’; ‘Lietzensee’; ‘Mittelsee’). Strains were assigned to species after Michaloudi *et al*.^27^ and Paraskevopoulou *et al*.^29^. Information on the different strains can be found in the Supplementary Information 1. Rotifers were raised in flasks with 200 ml of sterile and vitamin-supplemented Woods Hole Culture Medium (WC^45^) with saturating concentrations of *M*. *minutum* (SAG 243-1, Culture Collection of Algae, University of Göttingen, Germany) as food. Under these non-limiting dietary conditions *Brachionus* has a generation time of approx. 2 days. Prior to the experiments rotifers were sieved through a mesh (55 µm) and rinsed with sterile culture medium in order to separate them from their food.

The phytoplankton species *S*. *elongatus* (SAG 89.79), *M*. *minutum* (SAG 243-1) and *N*. *limnetica* (SAG 18.99) were used as food in the experiments because they differ fundamentally in their lipid composition (Table 2). Each species was cultured in a chemostat system filled with 700 ml of sterile modified WC medium with reduced inorganic nitrogen concentration (80 µmol L^−1^). They were cultured with a flow rate of 0.3 d^−1^ at 22 °C under continuous illumination of 100 µmol photons m^−2^ s^−1^. The carbon concentration $c$ of each algal chemostat monoculture was estimated from light extinctions ${OD}$ (800 nm; UV Shimadzu spectrophotometer, Duisburg, Germany) using pre-established calibration lines (light extinction versus carbon):

$$c[mgC{L}^{-1}]={OD}_{800nm}\times f,$$

where $f$ was 180, 120, and 140 for *S*. *elongatus*, *M*. *minutum*, and *N*. *limnetica*, respectively. For the experiments, carbon concentrations of respective food suspensions were adjusted by adding sterile WC medium.

### Experimental procedure

In the first population growth experiment, the six rotifer strains fed on two carbon concentrations (0.4 and 1.6 mg C L^−1^) of the three food sources in a full-factorial design with four replicates. The experiment was conducted in 6-well microtiter plates at 22 °C in the dark. In the beginning, 10 individuals were randomly chosen from the stock culture and pipetted into each well filled with 10 ml of the respective food suspension. At intervals of 24 h, living and dead rotifers in each well were counted and 10 live individuals were randomly picked and transferred into wells with newly prepared food suspensions. In the case that less than 10 individuals were alive all remaining were transferred. The experiment lasted for seven days. Microtiter plates were placed on a rocker (Bio-Rad, Double Rocker, Labnet International Inc., Woodbridge, NJ, USA) to reduce sedimentation of algal cells.

On a daily basis, for each replicate the intrinsic growth rate ($r$), the fecundity ($m$), and the probability of survival ($l$) per day ($t$) were calculated using the following equations:

$$r={\rm{ln}}({N}_{t})-{\rm{ln}}({N}_{t-1});$$

$$m=\frac{{H}_{t}}{{N}_{t-1}};$$

$$l=1-\frac{{D}_{t}}{{N}_{t-1}},$$

where ${N}_{t-1}$ is the initial number of individuals and ${N}_{t}$, ${H}_{t}$, and ${D}_{t}$ are the final numbers of individuals, of newly hatched individuals, and of dead, respectively, on consecutive experimental days. The population growth rate [d^−1^] of each replicate as well as fecundity [neonates ind^−1^ d^−1^] and the probability of survival [d^−1^] were calculated by averaging $r$, $m$, or $l$ of consecutive experimental days. The first experimental day was excluded from the calculations in order to consider time for rotifers to acclimate to the experimental conditions.

For the second population growth experiment, only five strains of the two rotifer species were available, as the ‘Mittelsee’ strain culture got lost. While our first experiment addressed strain-specific interactive effects of food quantity and biochemical food quality on the performance of *Brachionus*, in our second experiment we minimized the effect of food quantity to study particular food quality aspects by choosing a higher food concentration. The strains were provided with 3.2 mg C L^−1^ of *S*. *elongatus* supplemented with liposomes containing no or high amounts of cholesterol, resulting in dietary cholesterol concentrations of 0 and 10 µg (mg C)^−1^, respectively (N = 5). Liposomes were produced following the protocol described in Wacker and Martin-Creuzburg^17^. The experimental procedure and the calculation of the population growth rates were similar to the first experiment.

### Statistical analysis

The impact of food quantity, type of food algae, rotifer strains, and rotifer species on population growth rates, fecundity, and the probability of survival was analysed using three-way ANOVAs with rotifer strains nested within species. To meet assumptions of normality of residuals and homogeneity of variances, all population growth rates and fecundities were Box-Cox-transformed (λ = 1.5 and 0.66, respectively) according to Crawley^46^. Probabilities of survival were arcsine-transformed. Population growth rates of the cholesterol supplementation experiment were Box-Cox-transformed (λ = −2) to meet assumptions. A two-way ANOVA was applied with rotifer strains nested within species to analyse effects of cholesterol, species and strains on population growth rates. All statistical analyses were conducted using R^47^. The R package ‘ggplot2’^48^ was used for data visualization.

## Supplementary information

Supplementary Info 1, 2, and 3

## Data Availability

The experimental data generated and analysed during the current study are available from the corresponding author on reasonable request.
