# Supplementary material for: Fitness response variation within and among consumer species can be co-mediated by food quantity and biochemical quality
Source: Sci Rep. 2019 Nov 6;9:16126. doi: 10.1038/s41598-019-52538-2 (PMC6834596; doi:10.1038/s41598-019-52538-2)
Supplement: Supplementary file 1 — Supplementary Info 1, 2, and 3 [file 41598_2019_52538_MOESM1_ESM.pdf]

Title: Fitness response variation within and among consumer species can be co-mediated by food quantity and biochemical quality

Journal: Scientific Reports

Svenja Schälicke*, Johannes Teubner, Dominik Martin-Creuzburg, and Alexander Wacker

*Corresponding author: Svenja Schälicke, University of Potsdam, Am Neuen Palais 10, D-14469 Potsdam, Germany, svenja.schaelicke@uni-potsdam.de, Phone: + 49 331 977 1978

Supplementary Information 1 – Information on rotifer strains

Table S1 Information on strains of the rotifer species *Brachionus calyciflorus* s.s. and *Brachionus fernandoi*, which were used in the experiments. For speciation see Michaloudi et al. [1].

Species	Strain	Year, origin of isolation	Isolated or provided by	References
B. calyciflorus s.s.	IGB	unknown	K.-O. Rothhaupt	[2,3]
B. calyciflorus s.s.	USA	2001, Lake Michigan, Milwaukee, USA	M. Boraas, G. Fussmann	[4–6]
B. calyciflorus s.s.	Cornell	unknown	L. Becks	N/A
B. fernandoi	No.2484	2016, Pond near Raakow, Germany	M. Litwin	[6]
B. fernandoi	Lietzensee	2014, Lietzensee, Berlin, Germany	C. Schirmer	[6]
B. fernandoi	Mittelsee	2016, Mittelsee, Höhenland, Germany	S. Paraskevopoulou	[6]

References

1. Michaloudi, E. *et al.* Reverse taxonomy applied to the brachionus calyciflorus cryptic species complex: Morphometric analysis confirms species delimitations revealed by molecular phylogenetic analysis and allows the (re) description of four species. *PLoS One* **13**, 1–25 (2018).

2. Wacker, A. & Martin-Creuzburg, D. Biochemical nutrient requirements of the rotifer Brachionus calyciflorus: co-limitation by sterols and amino acids. *Funct. Ecol.* **26**, 1135–1143 (2012).

3. Schälicke, S., Sobisch, L., Martin-Creuzburg, D. & Wacker, A. Food quantity–quality co-limitation: Interactive effects of dietary carbon and essential lipid supply on population growth of a freshwater rotifer. *Freshw. Biol.* **64**, 903–912 (2019).

4. Fussmann, G. F., Ellner, S. P. & Hairston Jr, N. G. Evolution as a critical component of plankton dynamics. *Proc. Biol. Sci.* **270**, 1015–22 (2003).

5. Seifert, L. I. *et al.* Heated relations: Temperature-mediated shifts in consumption across trophic levels. *PLoS One* **9**, (2014).

6. Paraskevopoulou, S., Tiedemann, R. & Weithoff, G. Differential response to heat stress among evolutionary lineages of an aquatic invertebrate species complex. *Biol. Lett.* **14**, (2018).

Supplementary Information 2 – Susceptibilities to biochemical food quality differences

Table S1 Susceptibilities of population growth rates, fecundity and survival of *Brachionus calyciflorus* s.s. (*B.c.*) and *Brachionus fernandoi* (*B.f.*) to food quality. Six strains, three of each species, were provided with two quantities (0.4 and 1.6 mg C L^{-1}) of three unicellular algae. *Nannochloropsis limnetica* was used as reference food alga of high biochemical quality. Differences in the response to the food algae *Synechococcus elongatus* (*S.e.*) and *Monoraphidium minutum* (*M.m.*) compared to *N. limnetica* are provided as effect ranges. Negative values refer to negative effects of the alga as food compared to *N. limnetica*, while positive values refer to positive effects. The lower the value, the higher the susceptibility of the fitness measure to algal biochemical food quality differences. Values represent means ± standard deviation. For strains, the number of replicates was N = 4 with five exceptions in the population growth rates with N = 3 (IGB and Cornell fed 1.6 mg C L^{-1} *M. minutum*; No.2484, Lietzensee, and Mittelsee fed 0.4 mg C L^{-1} *S. elongatus*). For species, all replicates of the respective strains were included for the calculation, resulting in N = 72 for fecundity and survival and N = 70 and N = 69 for population growth rates of *B. calyciflorus s.s.* and *B. fernandoi*, respectively.

RANGES		Strains of *B. calyciflorus* s.s.			Strains of *B. fernandoi* sp. nov			Species	
		IGB	USA	Cornell	No.2484	Lietzensee	Mittelsee	*B.c.*	*B.f.*
**0.4 mgC L^{-1}**									
Population growth rate	*S.e.*	-0.77 (0.12)	-1.08 (0.12)	-0.85 (0.16)	-1.28 (0.46)	-0.85 (0.26)	-0.97 (0.40)	-0.90 (0.18)	-1.03 (0.38)
(d^{-1})	*M.m.*	-0.59 (0.21)	-0.07 (0.03)	-0.42 (0.11)	0.04 (0.11)	0.04 (0.02)	-0.15 (0.09)	-0.36 (0.26)	-0.03 (0.12)
Fecundity	*S.e.*	-0.98 (0.05)	-1.00 (0)	-0.98 (0.05)	-1.00 (0)	-0.84 (0.04)	-0.86 (0.10)	-0.98 (0.04)	-0.90 (0.09)
(neonates ind^{-1} d^{-1})	*M.m.*	-0.72 (0.24)	0.40 (0.05)	-0.84 (0.15)	-0.04 (0.16)	-0.25 (0.09)	-0.39 (0.18)	-0.39 (0.60)	-0.23 (0.20)
Probability of survival	*S.e.*	-0.54 (0.07)	-0.55 (0.10)	-0.61 (0.07)	-0.84 (0.13)	-0.65 (0.05)	-0.78 (0.13)	-0.57 (0.08)	-0.76 (0.13)
(ind^{-1} d^{-1})	*M.m.*	-0.44 (0.15)	0.07 (0.02)	-0.29 (0.12)	0.00 (0.07)	-0.02 (0.05)	-0.04 (0.05)	-0.22 (0.24)	-0.02 (0.06)
**1.6 mgC L^{-1}**									
Population growth rate	*S.e.*	-1.03 (0.11)	-1.05 (0.16)	-1.08 (0.10)	-1.57 (0.17)	-0.85 (0.15)	-0.92 (0.06)	-1.05 (0.12)	-1.11 (0.36)
(d^{-1})	*M.m.*	-1.01 (0.03)	-0.22 (0.12)	-0.49 (0.11)	-0.17 (0.09)	0.10 (0.07)	-0.13 (0.03)	-0.54 (0.36)	-0.07 (0.14)
Fecundity	*S.e.*	-0.94 (0.04)	-0.94 (0.03)	-0.99 (0.01)	-0.95 (0.05)	-0.97 (0.02)	-0.99 (0.01)	-0.96 (0.04)	-0.97 (0.03)
(neonates ind^{-1} d^{-1})	*M.m.*	-0.87 (0.15)	-0.15 (0.25)	-0.52 (0.20)	-0.43 (0.11)	-0.39 (0.10)	-0.46 (0.04)	-0.52 (0.36)	-0.43 (0.08)
Probability of survival	*S.e.*	-0.30 (0.11)	-0.22 (0.14)	-0.23 (0.12)	-0.78 (0.02)	-0.49 (0.06)	-0.38 (0.13)	-0.25 (0.12)	-0.55 (0.19)
(ind^{-1} d^{-1})	*M.m.*	-0.25 (0.09)	0.02 (0.03)	-0.14 (0.05)	0.04 (0.04)	-0.01 (0.02)	0.02 (0.02)	-0.12 (0.13)	0.02 (0.03)

Supplementary Information 3 – Ingestion rate experiment

Methods

Rotifers and algae were precultured as described in the main text.

For the ingestion experiment only five strains of the two rotifer species were available, as the 'Mittelsee' strain culture got lost. Each rotifer strain was supplied with an array of the same algae species and the same food concentrations as in the population growth experiment, but with 5 replicates per treatment. The experiment was run in 12-well microtiter plates, each filled with 4 ml of the respective food suspension. Before the experiment started all rotifers were put on the respective food in the respective concentration for 3 h to acclimate to the food conditions. At the start of the experiment, 10 rotifers per replicate were pipetted into a well. Individuals without eggs were picked to avoid changes in population size during the experiment. A control treatment without rotifers was set up to assess growth of algae during the experiment. All plates were placed in a dark climate chamber of 22 °C and on a rocker to reduce sedimentation of algal cells. An aliquot of the food suspension was taken to a flow cytometer (BD Accuri C6 Flow Cytometer, BD Biosciences, USA) equipped with BD Accuri C6 Software (BD Biosciences, USA) to measure the start concentration of the algal cells. The number of cells in 40 µl of 1 ml subsample was measured with a medium flow rate of 35 µL min^{-1}. Therefore, fluorescence intensities of cells were collected using the FL3 and FL4 channels with laser excitation wavelengths of 488 nm and 640 nm, respectively, and threshold values of 1000. Gates were set for each algae species to separate cell signals from sample background signals. After 24 h of grazing the final algal cell concentration per replicate was measured. Therefore, a subsample of 1 ml of each replicate was pushed through a mesh (55 µm) to separate the algal cells from the rotifers following flow cytometry measurements as described above. The respective carbon concentrations were recalculated using the carbon concentrations and the measured cell concentrations at the beginning of the experiment. By calculating the clearance rate F [µL ind^{-1} h^{-1}] for each rotifer strain, food suspension, and replicate an ingestion rate I [ng C ind^{-1} h^{-1}] was determined using the following equations:

$$F = \left(\ln\left[\frac{c_{start}}{c_{final}}\right] - \ln\left[\frac{x_{start}}{x_{final}}\right]\right) * \left(\frac{V}{N*t}\right),$$

$$I = F * \sqrt{c_{start} * c_{final}},$$

Where c_{start} and x_{Start} are the start concentrations [ng C L^{-1}] of grazing and control treatments, respectively, c_{final} and x_{final} the respective final concentrations [ng C L^{-1}], V is the experimental volume [µl], t is the time period rotifers grazed [h], and N is the number of individuals per replicate.

Influences of variables on ingestion rates were investigated using a three-way ANOVA with rotifer strains nested within species. Although using a log10(x+1)-transformation of the whole data the normality of residuals and the homogeneity of variances was improved, it could not completely be ensured. Such, however, was possible by separating the data into subsets: As our focus was explicitly on strain and species effects, we applied nested ANOVAs for each algae and food quantity separately in order to verify the results for strain and species effects found in the full model (the nested three-way ANOVA). All statistical analyses were conducted using R[1]. The R package 'ggplot2'[2] was used for data visualization.

Results

We analyzed ingestion rates in order to exclude ingestion of prey as an influencing factor for differences in population growth responses among strains or species. A nested three-way ANOVA confirmed expected general effects of food quantity (nested three-way ANOVA, factor food quantity, $F_{1,115} = 786.4$, $P < 0.001$) and food alga (factor food alga, $F_{1,115} = 119.9$, $P < 0.001$; Table S1) on ingestion. The ingestion rate neither differed among rotifer species (factor species, $F_{1,115} = 0.3$, $P = 0.60$; interaction, $F_{1,115} = 1.3$, $P = 0.29$), nor among strains (interaction, $F_{1,115} = 1.2$, $P = 0.28$; Fig. S1, Table S1). Single nested ANOVAs applied separately for each food quantity and each alga confirmed that all algae were ingested equally among strains ($P > 0.05$). This suggests, strains and species did not differ in their prey size preference. Effects of morphological prey traits, which could have influenced ingestion, were therefore excluded. Instead, we assume that differences in the nutritional quality of the prey species, such as their lipid contents, account for food algae effects on population growth, fecundity and survival responses of rotifer strains.

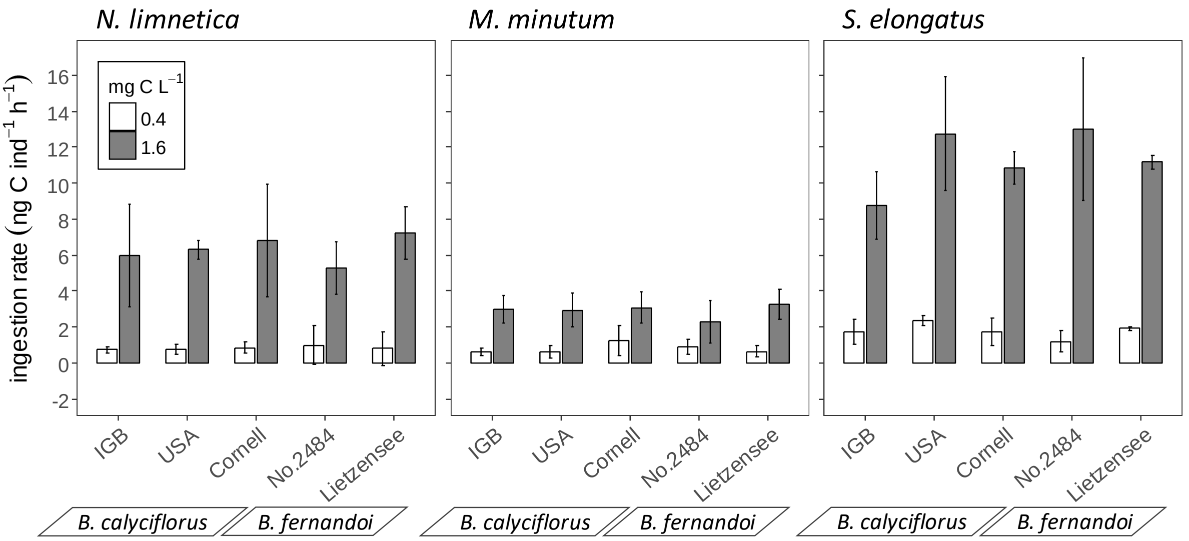

Fig. S1 Ingestion rates of five strains of the two rotifer species *Brachionus calyciflorus* s.s. ('IGB, 'USA', 'Cornell') and *Brachionus fernandoi* ('No.2484', 'Lietzensee') feeding on one of three unicellular algae species (*Nannochloropsis limnetica*, *Monoraphidium minutum* and *Synechococcus elongatus*), that differ in their biochemical quality. Each food algae was provided in two quantities (0.4 and 1.6 mg C L^{-1}). Values represent means ± standard deviations, number of replicates N = 5.

Table S1 Results of a three-way ANOVA using log-transformed ingestion rates of five different strains nested within the two rotifer species *Brachionus calyciflorus* s.s. (three strains) and *Brachionus fernandoi* (two strains). The five strains were provided with two quantities (0.4 and 1.6 mg C L^{-1}) of three unicellular algae (*Synechococcus elongatus*, *Monoraphidium minutum*, *Nannochloropsis limnetica*) of different biochemical quality.

	Ingestion rate (ng C ind^{-1} h^{-1})		
Independent variable(s)	*df*	*F*-value	*P*-value
Food quantity (FQ)	1, 115	786.4	**<0.001**
Food alga (FA)	2, 115	119.9	**<0.001**
Species (Sp)	1, 115	0.3	0.60
FQ × FA	2, 115	24.6	**<0.001**
FQ × Sp	1, 115	0.5	0.47
FA × Sp	2, 115	0.03	0.97
FQ × FA × Sp	2, 115	1.3	0.29
FG × FA × Sp × Strain	18, 115	1.2	0.28

References

1. R Core Team. R: A language and environment for statistical computing. R Foundation for Statistical Computing, Vienna, Austria (2018). https://www.R-project.org/

2. Wickham, H. ggplot2: Elegant graphics for data analysis. New York, NY: Springer-Verlag (2016). ISBN 978-3-319-24277-4, retrieved from http://ggplot2.org
